# *Plasmodium vivax* Biology: Insights Provided by Genomics, Transcriptomics and Proteomics

**DOI:** 10.3389/fcimb.2018.00034

**Published:** 2018-02-08

**Authors:** Catarina Bourgard, Letusa Albrecht, Ana C. A. V. Kayano, Per Sunnerhagen, Fabio T. M. Costa

**Affiliations:** ^1^Laboratory of Tropical Diseases, Department of Genetics, Evolution, Microbiology and Immunology, University of Campinas - UNICAMP, Campinas, Brazil; ^2^Laboratory of Regulation of Gene Expression, Instituto Carlos Chagas, Curitiba, Brazil; ^3^Department of Chemistry and Molecular Biology, University of Gothenburg, Gothenburg, Sweden

**Keywords:** malaria, *Plasmodium vivax*, genome, transcriptome, proteome

## Abstract

During the last decade, the vast omics field has revolutionized biological research, especially the genomics, transcriptomics and proteomics branches, as technological tools become available to the field researcher and allow difficult question-driven studies to be addressed. Parasitology has greatly benefited from next generation sequencing (NGS) projects, which have resulted in a broadened comprehension of basic parasite molecular biology, ecology and epidemiology. Malariology is one example where application of this technology has greatly contributed to a better understanding of *Plasmodium* spp. biology and host-parasite interactions. Among the several parasite species that cause human malaria, the neglected *Plasmodium vivax* presents great research challenges, as *in vitro* culturing is not yet feasible and functional assays are heavily limited. Therefore, there are gaps in our *P. vivax* biology knowledge that affect decisions for control policies aiming to eradicate vivax malaria in the near future. In this review, we provide a snapshot of key discoveries already achieved in *P. vivax* sequencing projects, focusing on developments, hurdles, and limitations currently faced by the research community, as well as perspectives on future vivax malaria research.

## Introduction

The last 10 years brought breakthroughs in biological research with the emergence of and improved accessibility to sequencing technology (Margulies et al., 2005). Nowadays, the increasingly available, user-friendly and less costly next generation sequencing (NGS) (Metzker, 2010) platforms are major technological contributions. Genomic exploration of organisms in greater detail through whole genome sequencing (WGS) is a reality. Additional valuable insights are coming from whole transcriptome (WTS) and proteome sequencing projects within the wider “omics” field, which start to shed light on multiple aspects of biology. Data integration within the “omics,” quantitative, functional and regulatory analysis - systems biology - is revolutionizing our understanding of the mechanisms of life. All these interconnected genomic, transcriptomic and proteomic platforms bring a new world of possibilities for hypothesis-driven research.

Genome sequencing projects revealed *Plasmodium* spp. genetic diversity and contributed to a better knowledge of parasite biology and host-parasite interaction (Hemingway et al., 2016). A significant number of fully sequenced and high quality *Plasmodium* spp. reference genomes are available (Carlton et al., 2008a; Dharia et al., 2010; Menard et al., 2010, 2013; Westenberger et al., 2010; Bright et al., 2012; Chan et al., 2012; Neafsey et al., 2012; Flannery et al., 2015; Winter et al., 2015; Hupalo et al., 2016; Pearson et al., 2016), and sequence curation efforts should continue. Today, the malaria research community ventures more and more into transcriptomics (Bozdech et al., 2008; Hoo et al., 2016; Zhu et al., 2016) and proteomics (Ray et al., 2016, 2017), areas to capitalize on for understanding *Plasmodium* spp. biology, especially the dynamics of RNA and protein expression and regulation through its complex multi-staged life cycle, in host and vector interaction contexts, and under different environmental selective pressures. Hence, the expertise provided by in-depth sequencing projects with clear data integration for understanding metabolic pathways in a systematic way is welcome by the parasite research community. Not only does it provide important pieces of information to understand different immune evasion and host invasion strategies, it is also a way to monitor and find new means to combat the rapidly increasing transmission of drug resistant parasites, and to identify molecular targets as starting points for effective vaccine development.

*Plasmodium vivax* malaria research has historically faced numerous technical adversities and has been largely neglected. This situation has led to a general lack of knowledge of *P. vivax* biology, and consequently impaired our capacity for making the best decisions on transmission control measures, and in the long run, for vivax malaria eradication. Currently vivax malaria is acknowledged as a disease that should no longer be neglected as it has been shown to result in considerable morbidity and mortality (Alexandre et al., 2010; Andrade et al., 2010; Lacerda et al., 2012a; Quispe et al., 2014; Rodriguez-Morales et al., 2015; Siqueira et al., 2015).

In this review, we show the main achievements on *P. vivax* biology accomplished based on the published genome, transcriptome and proteome sequencing projects. In particular, (1) the genome-wide comparative studies showing evolutionary relationships between parasites of the same genus, (2) the broad genetic diversity landscape within the *P. vivax* populations reported as to understand specific selection pressures (environmental and host/vector related) acting presently on parasite populations, (3) the more recent expression profile datasets and regulation mechanisms emerging from sensitive high-throughput WTS (RNA-seq) of *P. vivax* in different stages, and (4) the attempts to identify parasite metabolic pathways and antigens as possible diagnosis biomarkers through mass spectrometry (MS) based proteomics analysis of parasites and human host profiling from vivax malaria patient samples. Also, we present the main challenges encountered, remaining gaps and possible research avenues being explored and developed by the vivax malaria community.

## Vivax malaria: an overview

Human malaria infections can be caused by five different *Plasmodium* species. *P. falciparum* is considered the deadliest parasite, causing the most severe clinical outcomes, whereas *P. vivax* is the most geographically spread within densely populated regions, thus accentuating the socio-economic burden caused by the disease (Gething et al., 2012; WHO, 2015). Recently, vivax malaria has re-emerged in regions formerly considered malaria free (Severini et al., 2004; Kim et al., 2009; Bitoh et al., 2011). Worldwide, about 2.85 billion people have been estimated to be at risk of infection by *P. vivax* (Price et al., 2007; Guerra et al., 2010; Battle et al., 2012; Gething et al., 2012). Following the decline of *P. falciparum* infections, *P. vivax* is now the dominant malaria species in several endemic regions (Coura et al., 2006; Gething et al., 2012; Hussain et al., 2013; WHO, 2015), where reports show a higher incidence, especially in young children (Marsh et al., 1995; Williams et al., 1997; Price et al., 2007; Genton et al., 2008; Tjitra et al., 2008). Several clinical complications that were normally associated with *P. falciparum* infections have been reported for vivax malaria (Kochar et al., 2005, 2009a; Hutchinson and Lindsay, 2006; Baird, 2007; Barcus et al., 2007; Alexandre et al., 2010; Rahimi et al., 2014). The most observed include anemia (Haldar and Mohandas, 2009; Quintero et al., 2011), haemolytic, coagulation disorders, jaundice (Sharma et al., 1993; Erhart et al., 2004; Lacerda et al., 2004; Saharan et al., 2009) and acute respiratory distress syndrome (Anstey et al., 2002, 2007; Suratt and Parsons, 2006; Tan et al., 2008; Lacerda et al., 2012b; Lanca et al., 2012), followed by nephropathology (Chung et al., 2008), porphyria (Kochar et al., 2009b), rhabdomyolysis (Siqueira et al., 2010), splenic rupture (de Lacerda et al., 2007; Gupta, 2010), and cerebral malaria (Lampah et al., 2011; Tanwar et al., 2011). Vivax malaria during pregnancy causing spontaneous abortions, premature and low weight new-borns (McGready et al., 2004; Poespoprodjo et al., 2008) is another major health concern, challenging the pre-established view of *P. vivax* as a “benign” parasite (Mendis et al., 2001; Baird, 2007; Anstey et al., 2009; Mueller et al., 2009; Gething et al., 2012; Naing et al., 2014).

According to the World Health Organization (WHO) guidelines (WHO, 2015), the first line *P. vivax* chemotherapy is chloroquine (CQ) plus primaquine (PQ), the only approved drug targeting the latent parasite form (Beutler et al., 2007). In high transmission areas presenting cases of drug resistance, an artemisinin based combination therapy (ACT) is recommended (WHO, 2010). The constant increase and spread of anti-malarial drug resistance remains of great concern (Baird, 2004; de Santana Filho et al., 2007; Suwanarusk et al., 2007; Poespoprodjo et al., 2008; Russell et al., 2008; Tjitra et al., 2008; Price et al., 2009, 2014).

**Figure 1:**
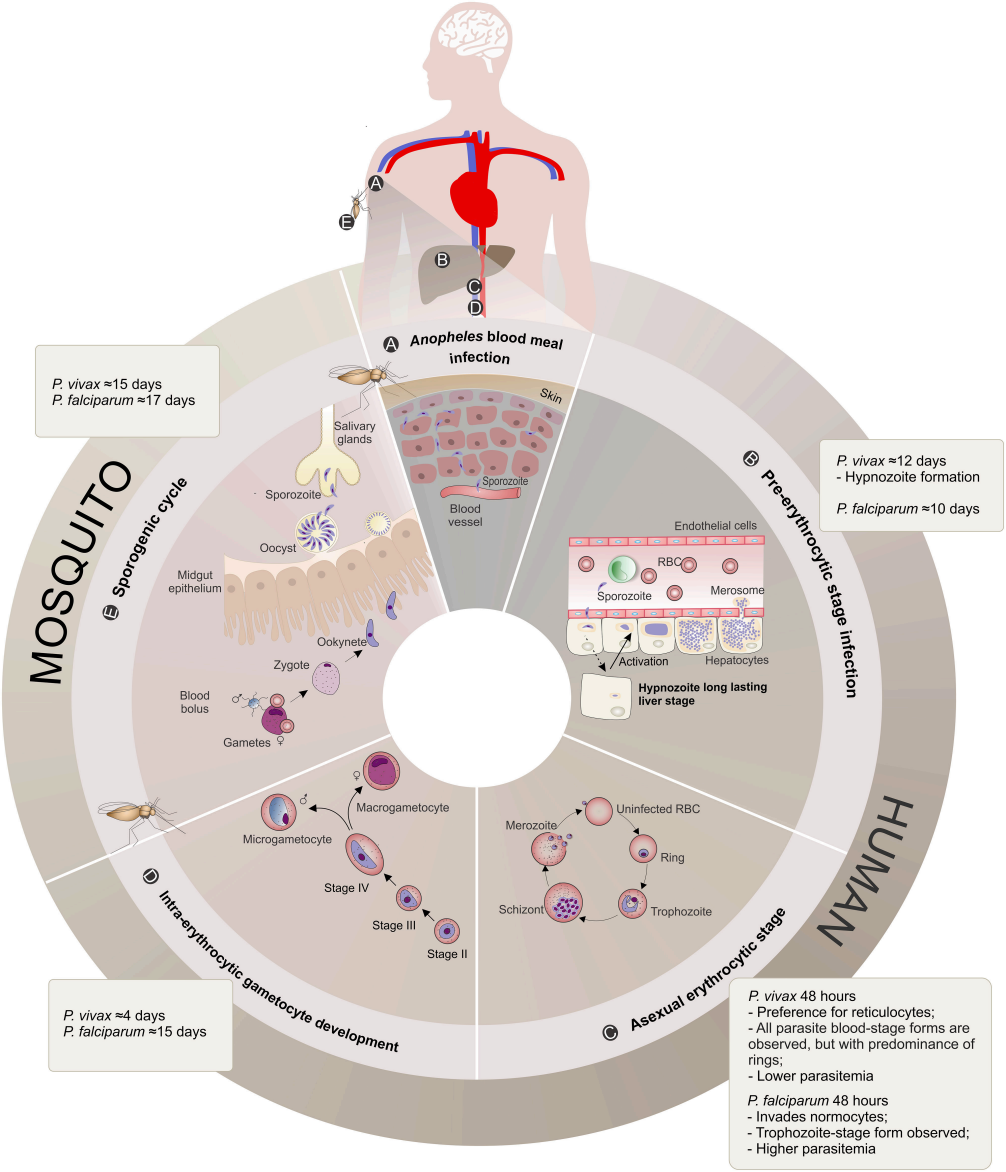
*Plasmodium vivax* and *Plasmodium falciparum* life cycle comparison. **(A)** *Anopheles* blood meal infection: Malaria infection occurs once a female *Anopheles* spp. mosquito inoculates *Plasmodium* sporozoites into the skin of the human host during its feeding (Kiszewski et al., 2004). The sporozoites eventually reach the bloodstream. **(B)** Pre-erythrocytic stage infection: The sporozoites are transported through the vascular system to the liver, where they migrate across Kupffer or endothelial cells and enter hepatocytes. When a hepatocyte is found and within a period of 10–12 days, sporozoites from both *Plasmodium* species form a parasitophorous vacuole membrane and differentiate into schizonts. The schizogony process involves thousands of mitotic replications giving rise to a high number of merozoites packed into merosomes, which are then released into the bloodstream. *P. vivax* sporozoites can also differentiate into dormant long lasting liver forms called hypnozoites that upon activation start schizogony with consequent release of merozoites into the vasculature, and consequently causing clinical relapses (Krotoski, 1985). **(C)** Asexual erythrocytic stage: During this 48 h stage, *P. vivax* merozoites have a preference for reticulocyte invasion, whereas *P. falciparum* invade normocytes. Upon invasion, the parasite promotes several alterations in these blood cells, enlarging and deforming them (Suwanarusk et al., 2004) and promoting the formation of caveola-vesicle like complexes (CVC) and cytoplasmic cleft structures (Barnwell et al., 1990), resulting in an appropriate environment for asexual schizogony in the bloodstream. The species-specific set of surface proteins produced by the parasites influences the proportion of different parasite stages observed in the patient's peripheral blood and are suggested to be linked to the different pyrogenic capacity, biomass and parasitemia levels presented by vivax or falciparum malaria patients. RBC, Red Blood Cell. **(D)** Intra-erythrocytic gametocyte development**:** A proportion of asexual parasites are known to undergo gametocytogenesis into round-shaped sexual micro- and macrogametocytes, quite early (4 days) after *P. vivax* infection compared to *P. falciparum* (15 days post-infection) (Pelle et al., 2015), before detection of any clinical symptoms. **(E)** Mosquito Stage: Gametocytes circulating in the bloodstream can be taken up by the next *Anopheles* blood meal and engage in the sexual cycle with release of male and female mature gametes, fertilization (zygote) and formation of motile ookinetes that cross the mosquito midgut epithelium and further mature into oocysts. The oocyst, a new asexual sporogonic replicative entity, generates and releases thousands of sporozoites. These sporozoites migrate and invade the salivary glands of the mosquito vector and can then be transmitted to a new human host, completing the complex life cycle of the parasite (Mueller et al., 2009).

*P. vivax* had an evolutionary path distinct from *P. falciparum*, being more closely related to *P. cynomolgi*, a sister taxon that infects Asian macaque monkeys (Duval et al., 2010; Liu et al., 2014; Luo et al., 2015). Probably as a consequence of such a unique evolutionary path, *P. vivax* shows unique biological features (Baird, 2007; Mueller et al., 2009; Gething et al., 2012) that distinguish it from *P. falciparum* (Figure 1):

1. Preference for invading reticulocytes (RTs) (Field and Shute, 1956), increasing their deformability, size, fragility and permeability (Kitchen, 1938; Suwanarusk et al., 2004; Handayani et al., 2009; Desai, 2014), which would allow *P. vivax* to evade the host immune system (del Portillo et al., 2004; Suwanarusk et al., 2004; Handayani et al., 2009) and maintain its characteristic low biomass parasitemia (Mueller et al., 2009).
2. Earlier production of sexual stages during the infection (Boyd and Kitchen, 1937), with a characteristic spherical shape seen in peripheral blood circulation even before the beginning of clinical symptoms, which might function as a reservoir to promote successful transmission to the mosquitoes (Boyd and Kitchen, 1937; Bousema and Drakeley, 2011).
3. Formation of hypnozoites, which remain in the liver in a latent state (Krotoski et al., 1982; Baird et al., 2007), and for which the reactivation mechanisms are still unknown (Mueller et al., 2009).

*P. vivax* research has been greatly restricted by lack of a reliable and reproducible *in vitro* system for long term culture (culturing efforts reviewed by Udomsangpetch et al., 2008; Noulin et al., 2013). This limitations are reflected both in the quantity and quality of functional assays performed in *ex vivo* short term cultures (Noulin et al., 2013). Furthermore, the alternative use of monkey-based studies with adapted strains of *P. vivax* (Beeson and Crabb, 2007; Panichakul et al., 2007) is of no easy access. Nevertheless, the available molecular tools (reviewed in Escalante et al., 2015) emerge as a valuable option to better estimate the prevalence and incidence of vivax malaria, its dynamics and differential contributions between host and vectors.

## Plasmodium vivax genomics

### Genome sequencing breakthroughs and projects

**Figure 2:**
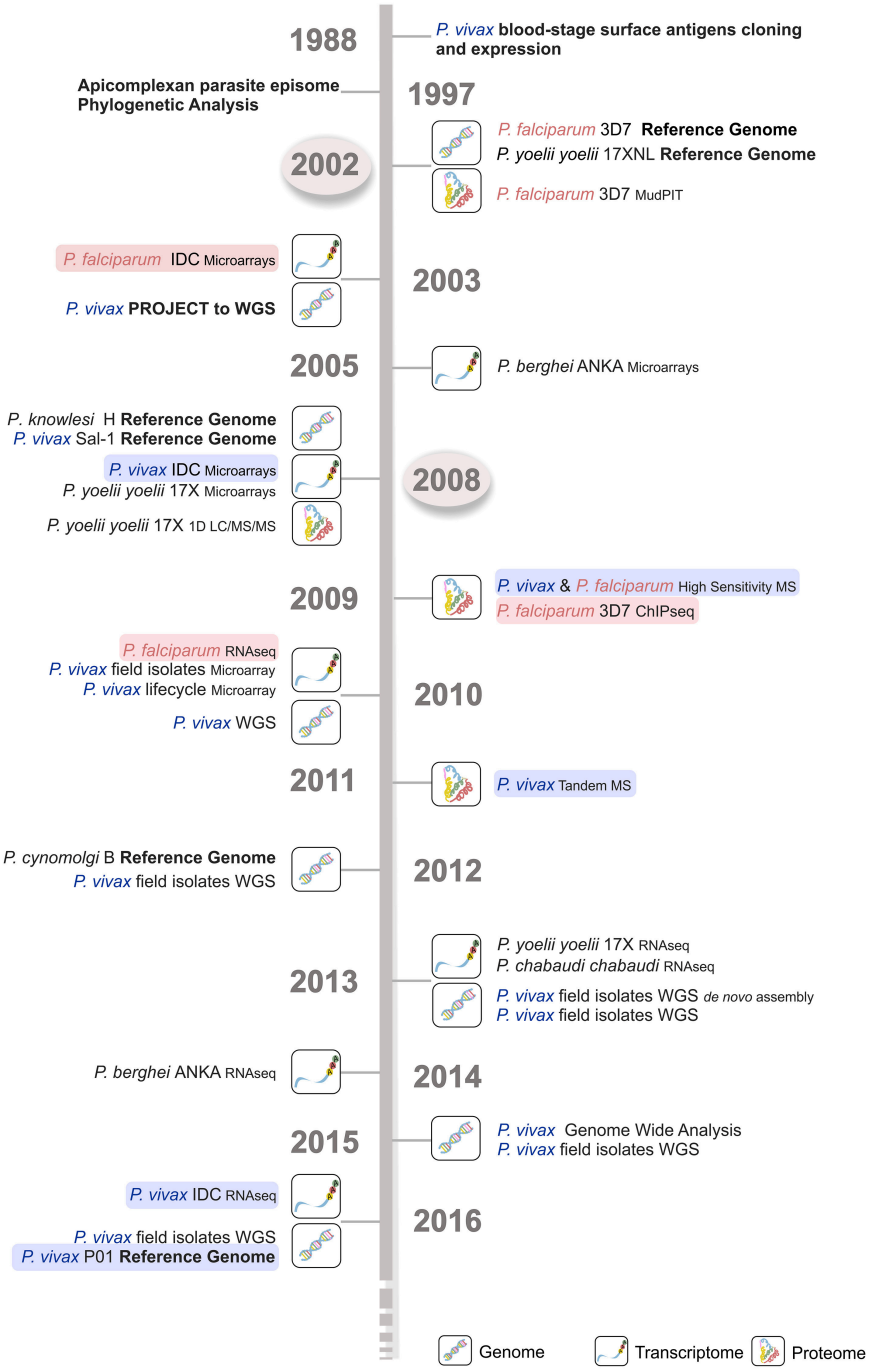
Time line of the major *Plasmodium* spp. sequencing projects. Genome, transcriptome, and proteome sequencing projects in the last four decades have contributed to our current parasite biology knowledge. *P. falciparum* (light red) and *P*. vivax (light blue) sequencing projects are shown, emphasizing the delay in time of around 5–6 years for the latter species. The first *P. falciparum* reference genome became available much earlier in 2002 (circled in light gray), then the corresponding *P. vivax* Sal-1 reference genome in 2008 (circled in light gray). WGS, whole genome sequencing; IDC, intraerythrocytic developmental cycle; MudPIT, multidimensional protein identification technology; MS, mass spectrometry; 1D LC-MS/MS, one-dimension liquid chromatography–mass spectrometry; RNA-seq, RNA sequencing; ChIP-seq, chromatin immunoprecipitation (ChIP) DNA sequencing.

In 2008, Carlton and colleagues achieved the complete generation, assembly and analysis of the first *P. vivax* WGS (Carlton, 2003; Feng et al., 2003; Carlton et al., 2008a), 6 years after the publication of *P. falciparum* reference genome (Gardner et al., 2002; Figure 2). The sequenced *P. vivax* Salvador-1 (Sal-1) strain came from a patient isolate from La Paz, close to El Salvador, and was passed to human volunteers, *Aotus* owl monkeys, and subsequently adapted to growth in *Saimiri* squirrel monkeys by mosquito and blood infection during the 1970s (Collins et al., 1972). Only later, it was possible to extract enough genomic DNA (gDNA) for whole genome shotgun sequencing (WGSS) and *de novo* assembly (Carlton et al., 2008a). Some technical hurdles were overcome, notably that the genome GC content of the parasite and laboratory host were similar and little information was available for the squirrel monkey gDNA sequence to exclude possible contamination (Carlton, 2003).

The constraints initially faced by these studies to acquire sufficient gDNA of acceptable quality for WGS were alleviated by the use of NGS platforms. These allowed direct sequencing of the first patient *ex vivo* isolate in 2010 (Dharia et al., 2010) without the need for *in vitro* propagation (Figure 2) or use the of more traditional and laborious cloning and expression approaches (del Portillo et al., 1988). Since then, sequencing technology sensitivity has increased while required genetic material amounts have decreased, leading to lower costs. As a result, NGS is now a portable benchtop technology much closer to endemic areas. Parasite DNA can be enriched in low parasitemia blood samples and human contamination eliminated (Auburn et al., 2013). To achieve an effective preparation of *P. vivax* field isolates for WGS, a technique combining a CF11-based human lymphocyte filtration and the short-term *ex vivo* culture for schizont maturation was optimized and is currently applied with success in several labs set in endemic areas. An alternative approach to preventing host contamination and enriching samples in *P. vivax* DNA is the direct sequencing of parasite gDNA by hybrid selection (Melnikov et al., 2011). This sequencing methodology has allowed the first worldwide characterization of *P. vivax* isolates (Dharia et al., 2010; Menard et al., 2013).

Recently, several patient isolates from Peru, Madagascar, Malagasy, Cambodia, and a Belem monkey adapted strain were sequenced coupled with the resequencing of the Sal-1 strain (Carlton et al., 2008a) using whole genome capture (WGC), WGS and NGS techniques (Dharia et al., 2010; Bright et al., 2012; Chan et al., 2012; Neafsey et al., 2012; Winter et al., 2015; Figure 2). In 2016, major WGS analysis studies were published, including one sequencing 195 *P. vivax* genomes including 182 new high depth and quality sequences from clinical isolates from 11 different countries worldwide (Hupalo et al., 2016) and 13 already published sequences from both clinical isolates and monkey-adapted laboratory lines. In addition, 70 Cambodian *P. vivax* isolates, further compared to 80 *P. falciparum* isolates from the same region (Parobek et al., 2016) were sequenced, considerably augmenting the genomic data available to the malaria community (Luo et al., 2015; Hupalo et al., 2016; Parobek et al., 2016; Figure 2). Expectations are that these data will bring great advances in population genetics at a genome scale and that comparative genomic analysis (CGA) will function as a powerful method for parasite evolution hypothesis generation and testing (Feng et al., 2003).

**Figure 3:**
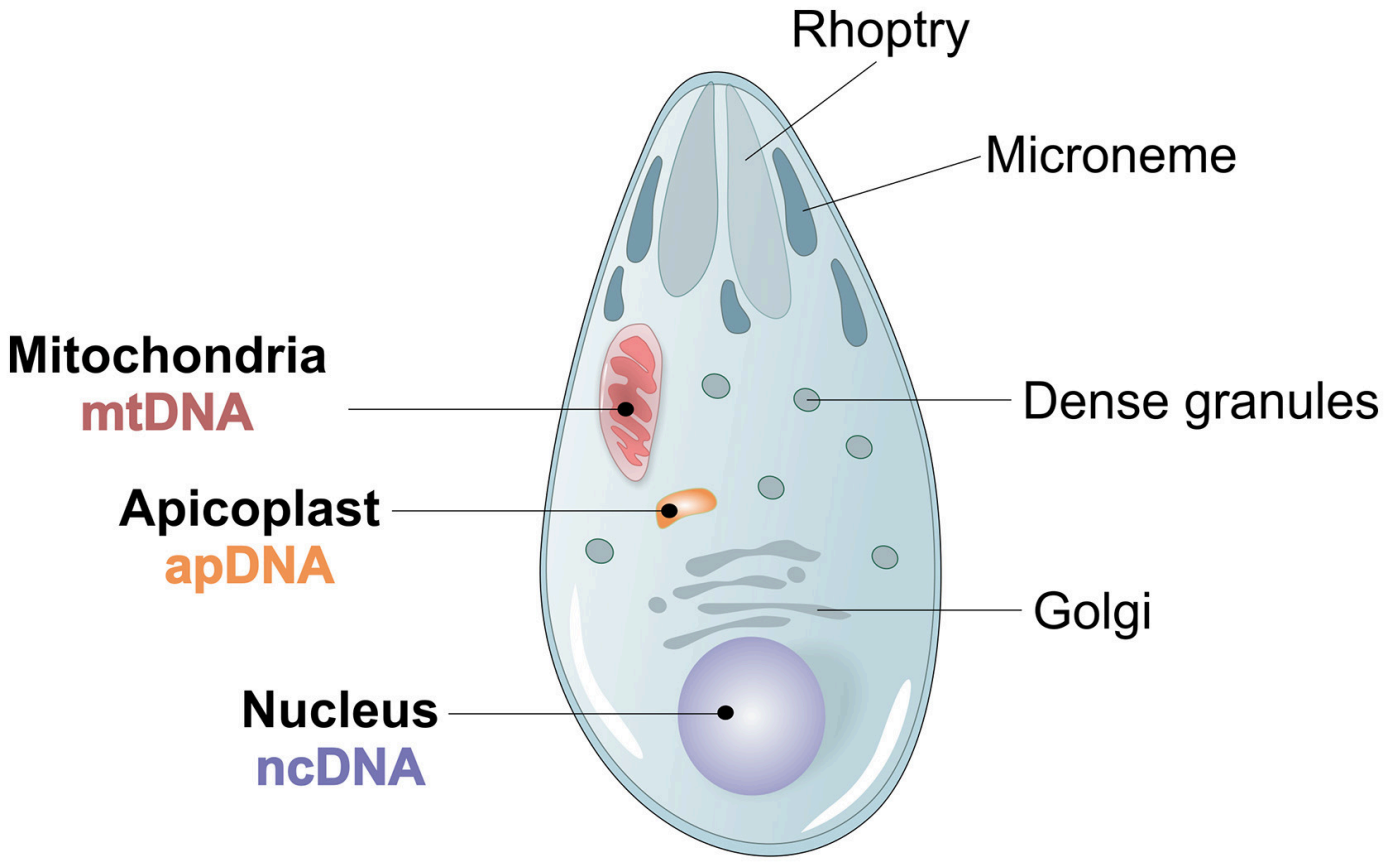
*Plasmodium* spp. morphology and genome architecture. Illustration showing the principal characteristics of Apicomplexan *Plasmodium* spp. parasite morphology, with its 3 parasite genomes, nuclear (ncDNA), mitochondrial (mtDNA) and apicoplast (apDNA).

Recently, a newly assembled and annotated reference genome named *P. vivax* P01 was published (Auburn et al., 2016a; Figure 2). The data was generated by using high-depth Illumina® sequencing from a Papua Indonesia patient isolate (Auburn et al., 2016a). For assembly, manual curation and comparison between data sets, the draft assemblies for the *P. vivax* isolates C01 (China) and T01 (Thailand) were used (Auburn et al., 2016a). The higher degree of assembly of these three genomes (~11 times) largely exceeds the Sal-1 reference genome (Carlton et al., 2008b; Auburn et al., 2016a). Not only was there an enhancement on scaffolds assembly with reduction from 2,500 unassembled scaffolds in Sal-1 to 226 in P01 reference genomes, as an improvement of the subtelomeric regions scaffold assembly. Therefore, annotation was also improved with the attribution of functions to 58% against the 38% in Sal-1 in a total of 4,465 core genes, defined as genes 1:1 orthologous between *P. vivax* P01 and *P. falciparum* 3D7 strains (Auburn et al., 2016a; Figure 3 and Table 1). The improvements in assembly and annotation quality in this sequencing project contributed to the generation of this very important new resource to study vivax malaria.

**Table 1:** *Plasmodium vivax* nuclear, mitochondrial and apicoplast genome features.

**Genomes**	***P. vivax*** **reference genomes**	
	**Sal-1 strain**	**P01 strain**
**NUCLEAR**		
Assembled size (Mb)	26.8	29.0
GC content (%)	42.3	39.8
Total n° of genes^*^	5,433	6,642
VIR protein	346	1,212
PvHIST protein, unknown function	64	84
PvTRAg protein	34	40
PST-A protein	11	10
PvSTP-1 protein	9	10
ETRAMP	10	9
RBP	9	9
Other unknown function exported proteins	23	447
**MITOCHONDRIAL**		
Assembled size (bp)	5,990	5,989
GC content (%)	30.5	30.5
**APICOPLAST**		
Assembled size (Kb)	5.1^**^	29.6
GC content (%)	17.1	13.3
N° of genes	0	30

*Table summarizing the P. vivax nuclear, mitochondrial and apicoplast genome features, both for Sal-1 stain (Carlton et al., 2008a) and the newly published P01 strain (Auburn et al., 2016a), including assembly size, GC content, total number of predicted genes (not including non-coding RNA genes) and subtelomeric most abundant multigene families (adapted from Auburn et al., 2016a)*.

* *Total number of genes include identified partial genes and pseudogenes*;

** *Published assembly of Sal-1 apicoplast reference partially sequenced; Mb, megabases; Kb, kilobases; bp, base pairs; VIR, variable interspersed repeat multigene family proteins; PvHIST, P. vivax Plasmodia Helical Interspersed SubTelomeric multigene family proteins (also named as Pf-fam-b) and RAD protein (Pv-fam-e); PvTRAg, P. vivax tryptophan-rich antigen family proteins, also named as Pv-fam-a and tryptophan-rich antigens; PST-A, lysophospholipase; PvSTP-1, P. vivax STP1 protein; ETRAMP, early transcribed membrane protein; RBP, reticulocyte binding protein*.

### Genome architecture and evolution

The biggest landmark achieved with the WGS of *Plasmodium* spp. was data acquisition to perform alignments and construct synteny maps with improved annotation, i.e., identifying conserved order of genetic loci along chromosomes (Ureta-Vidal et al., 2003). Through CGA one can now understand how the key forces that shape the evolutionary processes of organisms (mutation, selection, and genetic drift) came into play, by assuming that the genomes analyzed share a common ancestor (Frazer et al., 2003). For instance, CGA has allowed us to understand the integration of the apicoplast into apicomplexan species (Kohler et al., 1997) and to identify genes and their target signals transferred from the second endosymbiotic event into *Plasmodium* (Foth et al., 2003). Furthermore, CGA based on the *P. falciparum* and *P. yoelii yoelii* WGSs was the first such comparison between tropical pathogens of the same genus (Carlton et al., 2002; Feng et al., 2003), suggesting that chromosomal reshuffling might have been involved in speciation inside the *Plasmodium* genus.

Three different genomes, one nuclear, one mitochondrial and one apicoplast, characterize *P. vivax* (Figure 3). The ~26.8 megabases (Mb) nuclear genome is distributed among 14 chromosomes and has 42.3% GC average content, although presenting an isochore structure with high GC content in most internal chromosome regions, interspersed by high AT stretches mainly in subtelomeric regions (Carlton, 2003; Carlton et al., 2008a; Taylor et al., 2013; Table 1). The significance of *Plasmodium* spp. isochore structure remains unknown (Eyre-Walker and Hurst, 2001), however GC-rich genes evolve faster than AT-rich subtelomeric genes (Carlton, 2003; Carlton et al., 2008a). The codon usage in *P. vivax* is balanced (N_c_~54.2), with less biased use of the 61 codons (Carlton et al., 2008b; Yadav and Swati, 2012; Cornejo et al., 2015). In particular, gene location in telomeric, centromeric or chromosome interstitial regions was recently shown to present different codon biased compositions (Cornejo et al., 2015). Genes showing the highest codon usage bias encode housekeeping proteins, the most highly expressed. Interestingly, genes in this category also include the Pf/Pv-fam family putatively responsible for antigenic variation and associated with early gametocyte formation (Cornejo et al., 2015). These patterns have led to speculation regarding the strong influence of the mutation rate on local nucleotide (nt) composition. This may indicate dramatic variance in gene expression potential and profiles, and that genes encoded in such regions could intervene in the specific pathophysiology of *P. vivax* malaria (McCutchan et al., 1984; Carlton et al., 2008b; Cornejo et al., 2015). As previously observed for other organisms, *P. vivax* DNA enriched in CpG motifs stimulates the Toll-like Receptor 9 (TLR9) increasing the host inflammatory response (Parroche et al., 2007), previously thought to be mainly caused by hemozoin toxicity alone. Given that *P. vivax* has a higher GC content, a greater TLR9 activation upon parasite DNA uptake by the host has been hypothesized (Anstey et al., 2009), potentially contributing to the increased pyrogenicity of this parasite (Karunaweera et al., 1992; Hemmer et al., 2006).

With reservations for sequence misassembly, it was estimated that the *P. vivax* parasite has around 5,400 genes (Carlton et al., 2008a; Table 1), of which a large majority was found to be orthologous between several *Plasmodium* spp. (CGA on human parasites *P. vivax and P. falciparum*, primate parasites *P. cynomolgi* and *P. knowlesi* and the rodent parasites *P. yoelii. yoelii, P. chabaudi*, and *P. berghei*; Carlton et al., 2008a). Orthologous genes identified had conserved genome positions with no genome breakage hot spots (Carlton et al., 2008a). Specifically, the more closely related primate species *P. cynomolgi* and *P. knowlesi* (Glazko and Nei, 2003) showed a higher degree of conservation throughout the 14 chromosomes (Cornejo et al., 2015) with exception for multigenic regions, where frequent microsyntenic breaks were identified (Carlton et al., 2008b). Calculation of substitution rates at synonymous (dS) vs. non-synonymous (dN) sites has allowed estimation of the relative importance of selection and genetic drift (dN/dS) throughout *P. vivax* evolution. Although a great difference between average dS and dN values was observed in ~3,300 high-confidence *P. vivax*/*P. knowlesi* orthologous gene pairs, these values correlate within and between syntenic regions of chromosomes of the two species. Carlton and colleagues have suggested heterogeneous mutation rates across the genome (Carlton, 2003). In *Plasmodium*, genes encoding membrane-anchored, transmembrane, cell adhesion, exported proteins or extracellular proteins with signal peptide motifs, most of them in direct contact with the host defense system, evolve relatively faster than the ones encoding housekeeping proteins (Carlton et al., 2008a). Selective constraint analysis between the highly conserved *P. vivax*/*P. cynomolgi* genomes reported a minority of genes (<2%) as positively selected and consequently evolving quicker than others with heavily constrained functions (Carlton et al., 2008a). Therefore, it appears that evolutionary rates have been greatly influenced by host-parasite interactions leading to a high degree of variation between different gene classes in *Plasmodium* (Carlton et al., 2008a; Joy et al., 2008).

It has been suggested that *P. vivax* had an African origin (Koepfli et al., 2015; Winter et al., 2015) and subsequently spread through the world (Culleton et al., 2011; Liu et al., 2014). The recently analyzed *P. vivax* samples were gathered in two main groups, Old and New World (Hupalo et al., 2016). Today the diversity between Old World *P. vivax* and *P. falciparum* samples is significantly higher. This is indicative of a large effective population size in *P. vivax* that shows signs of population specific natural selection, where *P. vivax* is constantly adapting to human host and mosquito vector regional differences, and more recently, to antimalarial drug pressures (Neafsey et al., 2012; Winter et al., 2015; Hupalo et al., 2016; Parobek et al., 2016). These results were also seen at the local level for a high number of *P. vivax* samples from the Asian-Pacific region (Brazeau et al., 2016; Parobek et al., 2016; Pearson et al., 2016) and, in a smaller scale, in South American samples (Winter et al., 2015). The non-uniform distribution of the single nucleotide polymorphisms (SNPs) in *P. vivax*, clearly enriched at subtelomeric regions, is being interpreted as a result of local high recombination rates. This leads to the hypothesis that selection not only acts on genes, but also has been shaping the *P. vivax* genomic architecture. Natural selection can thus act faster on these regions where the generation of antigenic variation occurs and also have a direct influence on genome architecture and consequently, impact *P. vivax* adaptation (Cornejo et al., 2015). Further, genome-wide analysis of *P. vivax* genetic diversity points at interesting evolutionary implications. For instance, the *P. vivax* genome is generally under very strong constraint with negative selection, and only very few genes are being positively selected (Cornejo et al., 2015; Parobek et al., 2016). Such results suggest a longer evolutionary history of primate infection (Liu et al., 2014; Luo et al., 2015), clearly closer to other monkey-infecting *Plasmodium* spp. (Carlton et al., 2013) than to *P. falciparum*, making it more adapted to survive and replicate in primate hosts.

These results have supported the use of monkey malaria *Plasmodium* spp. as models for the adaptation of *P. vivax* parasite populations (Chan et al., 2015), Moreover, amplification in monkey hosts of patient isolates from several endemic areas (Brazil, North Korea, India, and Mauritania) (Galinski and Barnwell, 2012) was crucial to collect enough high-quality gDNA for WGS (Neafsey et al., 2012; Carlton et al., 2013). However, analysis of these sequences should take into account that the parasites isolated from vivax malaria patients were passed through multiple infection cycles and were adapted to grow in *Saimiri boliviensis* monkeys (Carlton et al., 2008b). Host switching and adaptation to a new *in vivo* immune environment could affect mutation rates, and consequently the degree of *P. vivax* sequence variation, expression profiles and multiclonal complexity of parasite populations (Hester et al., 2013). Nevertheless, the study of Chan and colleagues proved that research using *P. vivax* monkey-adapted strains resulted in useful data, however data interpretation should be done with caution, given that these strains are not always genetically homogeneous (Chan et al., 2015). Overall, WGS reveals specific differences within human malaria *Plasmodium* spp. which should be taken into account in future interventions (Carlton et al., 2008a; Pain et al., 2008; Frech and Chen, 2011; Tachibana et al., 2012; Hester et al., 2013; Cornejo et al., 2015; Winter et al., 2015; Hupalo et al., 2016; Parobek et al., 2016; Pearson et al., 2016).

## Plasmodium vivax transcriptomics

### Whole transcriptome sequencing projects

The first great effort into this parasite's transcriptome profile immediately followed the publication of *P. vivax* Sal-1 reference genome (Figure 2). By using a customized microarray platform of their own design, Bozdech et al. directly accessed *P. vivax* mRNA levels throughout 48 h intraerythrocytic developmental cycle (IDC) of three distinct isolates (Bozdech et al., 2008). Expression data analysis identified stage-specific differential expression of certain groups of genes. These genes were predicted to encode proteins with a role in parasitic development, virulence capacity and/or host-parasite interaction. In the following years, several microarray platform studies were published (Bozdech et al., 2008; Westenberger et al., 2010; Boopathi et al., 2013, 2014; Figure 2). Supporting the previous findings of Bozdech et al. on transcript levels at different lifecycle stages, Westenberger and colleagues described dramatic changes in messenger RNA (mRNA) co-expression of various genes predicted to be involved in developmental processes, suggesting that they could modulate parasitic progress through its different stages (Westenberger et al., 2010). In the 5′ region of co-expressed genes conserved motifs across *Plasmodium* spp. were identified as possible sites for regulatory protein binding with important roles in stage specific transcriptional regulation. Several multigene families and genes predicted to encode exported proteins displayed synchronized transcription in *P. vivax* sporozoites, but no difference (different gene set or mRNA levels) was observed between parasites showing capacity for hepatocyte invasion or hypnozoite development (Westenberger et al., 2010). Boopathi et al. reported a positive correlation of Natural Antisense Transcripts (NATs) with sense transcripts level, indicating that differing sense/antisense transcript ratios are involved in differential regulation of gene expression in diverse clinical conditions (Bozdech et al., 2008; Westenberger et al., 2010; Boopathi et al., 2013, 2014).

Very recently, RNA-seq was successfully applied to re-sequence two *P. vivax* isolates (Bozdech et al., 2008) throughout their IDC (Zhu et al., 2016; Figure 2). RNA-seq is more sensitive than gene expression microarrays as it detects expression nuances. In addition, RNA-seq includes all transcripts and not only the ones previously characterized present on microarray slides. The IDC transcriptome map produced by the high-resolution Illumina® HiSeq platform allows a better comprehension of the regulation behind the parasite gene expression profiles for specific biological functions. Strand-specific RNA-seq was just published for three isolates from Cambodian vivax malaria patient before anti-malarial treatment, and transcripts were assembled *de novo* revealing homogenous parasite gene expression profile regardless of the proportion of different stages (Kim et al., 2017). These results contrast with the gene expression pattern reported before (Bozdech et al., 2008; Zhu et al., 2016) and with data for sporozoites isolated from salivary glands of infected Colombian mosquitos (Kim et al., 2017). The authors recognize the fact that the patient isolates have multi-staged parasites, where one asexual stage might be transcriptionally more active than the others and, irrespective of its proportion, will deliver most transcripts, homogenizing the final expression patterns observed (Kim et al., 2017).

### Differential expression and regulation

*Plasmodium* parasites do not have the typical eukaryotic transcriptional apparatus, presenting a poor repertoire of transcription associated proteins (TAP) somewhat similar among *P. falciparum, P. vivax*, and *P. knowlesi*, but with a significant range of putative regulatory sequences in the genome. This raises the idea of a dynamic, complex and non-standard gene expression regulation system (Carlton et al., 2008a; Boopathi et al., 2013, 2014; Adjalley et al., 2016). From the microarray and RNA-seq studies, some of the *P. vivax* transcriptome architecture characteristics have emerged. Throughout IDC of *P. vivax* (and other *Plasmodium* spp.), the overall timing of gene expression profiles does not differ significantly between strains and isolates. Hence, transcriptional differences or variations observed between species might reflect distinct evolution paths (Bozdech et al., 2008). Species-specific gene expression patterns in members of multigene families may mirror separate environmental challenges and multiple and varied host-parasite interactions faced by the parasites (Bozdech et al., 2008; Westenberger et al., 2010; Boopathi et al., 2013; Zhu et al., 2016). Considerable changes were found on orthologous genes (30% divergence) between *P. falciparum* (Bozdech et al., 2003; Le Roch et al., 2003) and *P. vivax* (Bozdech et al., 2008; Westenberger et al., 2010; Zhu et al., 2016). Expression of these genes peaks at different moments and possibly contributes to a specific timing reflecting species-specific biological functions. Among these genes are those related to host-parasite interactions, red blood cells (RBC) invasion, early intraerythrocytic development and antigenic variation. *P. vivax* antigen-presenting gene families (e.g., *vir* and *trag* genes) are activated mainly at the schizont-ring stage transition, indicative of different transcriptional control between the two species and, once more, underscoring the marked biological distinctions between the two most important human malaria parasites (Bozdech et al., 2003, 2008). In addition, contrary to what was seen for *P. falciparum* (Bozdech et al., 2003), *P. vivax* seems to have an enhanced capacity to adapt to its host and modify its virulence factors, as shown by its extensive intra-isolate gene expression diversity (Bozdech et al., 2008). RNA-seq data analysis revealed a transcriptional hotspot of *vir* genes on chromosome 2, suggesting them as important for immune evasion mechanisms. Structurally, *P. vivax* genes present unusually long 5′ untranslated regions (UTRs) and multiple transcription start sites (TSSs) (Zhu et al., 2016; Kim et al., 2017). Supporting previous observations, Hoo et al. published the first integrated transcriptome analysis for six *Plasmodium* spp. showing that conserved syntenic orthologs present early parasitic stage transcriptional divergence, where expression patterns follow the corresponding mammalian hosts (Hoo et al., 2016) and changes in important putative transcriptional regulators might explain the observed transcriptional diversity. Conversely, orthologs presenting similar transcriptional profiles across all *Plasmodium* spp. might be responsible for conserved and crucial functions (Hoo et al., 2016).

**Figure 4:**
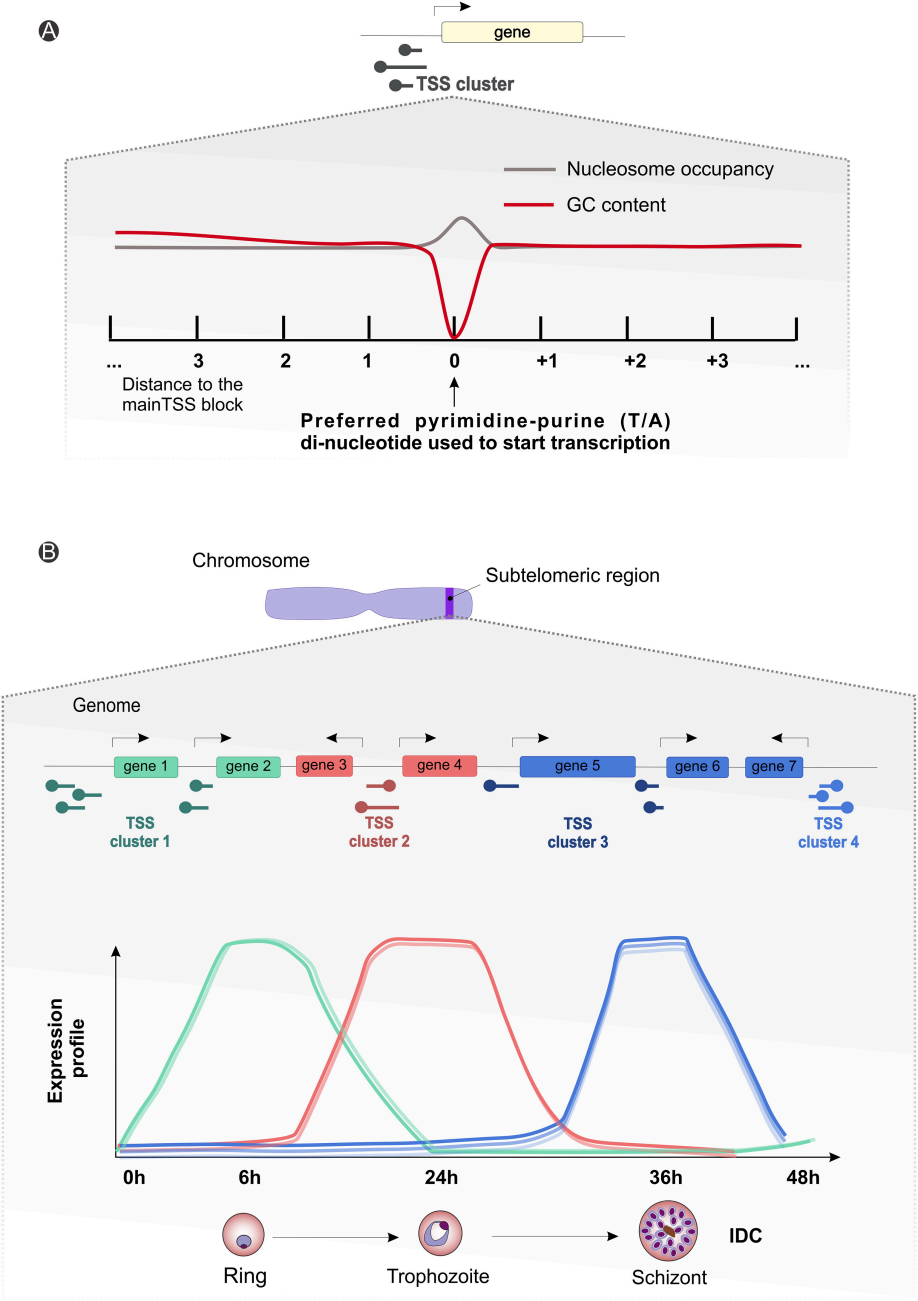
*Plasmodium* spp. gene expression and regulation dynamics. **(A)** Scheme illustrating the average observed nucleotide content and nucleosome occupancy surrounding the transcriptional start site blocks (black round tip arrow) controlling gene expression, with a preferential pyrimidine-purine (T/A) di-nucleotide usage (gray line) and a drop-in guanine-cytosine (GC) content (red line) around the starting site. **(B)** Generic representation showing an amplification of the suggested subtelomeric (blue strip on the chromosome drawing) transcriptional start site clusters (round tip arrows) dynamics for differential gene expression during parasitic intraerythrocytic developmental cycle (~48 h). The graph displays arbitrary subtelomeric gene expression profiles under different TSS clusters control, during ring (green, peak at 6 h), trophozoite (red, peak at 24 h) and schizont (blue, peak at 36 h) stages. IDC, intraerythrocytic developmental cycle; TSS, transcriptional start site.

The importance of clusters of TSSs, the significance of long 5′ vs. shorter 3′ UTRs, alternative splicing events and the profusion of non-coding RNAs (ncRNAs), in a context of genome-wide different GC content, is starting to be characterized (Zhu et al., 2016; Kim et al., 2017). Different sequence motifs have already been associated with stage specific expression regulation (Westenberger et al., 2010), and recently *Zhu* and colleagues observed a substantial variation of the 5′ UTR owing to differential selection of TSS, although these phenomena do not seem to be involved in regulation of the asexual parasite cycle. *P. falciparum* transcriptome analyses reveal uniform usage of several clusters of TTSs, but in a differentiated way (Figure 4A; Watanabe et al., 2002; Lenhard et al., 2012; Adjalley et al., 2016). Such sites are spaced along the genome, indicative of dispersed patterns of transcription both in coding and intergenic regions (Watanabe et al., 2002; Lenhard et al., 2012; Adjalley et al., 2016). With a broad bidirectional promoter sequence possibly controlled by multiple regulatory elements, also characteristic of other eukaryotic species, the architecture and sequence properties of *Plasmodium* spp. chromosomes can play an important role in transcription initiation and regulation by means of chromatin remodeling (Watanabe et al., 2002; Lenhard et al., 2012; Adjalley et al., 2016). Moreover, as for *P. falciparum, P. vivax* shows a cycling transcription pattern throughout the IDC (Bozdech et al., 2003; Le Roch et al., 2003), with stage-specific regulation of transcription initiation events, well correlated with gene expression levels (Figure 4B; Siegel et al., 2014; Adjalley et al., 2016).

Alternative splicing as a mechanism of producing transcriptome variability was reported for *P. falciparum* with a role in parasite sexual development (Iriko et al., 2009; Otto et al., 2010; Sorber et al., 2011), contrasting with the scarcity reported until now for *P. vivax* (Zhu et al., 2016). However, the few splicing events identified are linked to a late schizont stage, suggesting its significance at the gene function level (Zhu et al., 2016). Recently, Kim and colleagues described a high percentage of *P. vivax* genes encoding multiple, often uncharacterized, protein-coding sequences (Kim et al., 2017). In addition to long 5′ UTRs, many of the isoforms significantly differ in their 5′- or 3′-UTRs, possibly as a result of differential exon splicing. *P. vivax* may use such protein isoforms in transcription and/or translation regulation mechanisms. Importantly, examples of such cases support the idea that even for well-described genes conferring drug resistance, novel isoforms can be revealed through transcriptomic data analysis, which could aid in decoding molecular mechanisms responsible for antimalarial drug resistance (Hoo et al., 2016; Kim et al., 2017).

All *P. vivax* transcriptomic data allowing for ncRNAs identification reveal the presence of these RNAs at high levels, notably in the antisense direction (Zhu et al., 2016; Kim et al., 2017). In 2013, the NATs identified in *P. vivax* directly isolated from infected patients with different malaria complications by genome-wide transcriptome analysis using customized high density tiling microarray sequencing technology (Boopathi et al., 2014), suggested a possible role in differential regulation of *P. vivax* gene expression. The involvement of distinct types of transcriptional machinery and different mechanisms, such as transcriptional run-through, bidirectional and antisense-specific promoters, produces a varied landscape of differential regulation observed for *P. vivax* from malaria patients in diverse clinical conditions (Boopathi et al., 2013). Surprisingly, the extensively investigated micro RNAs (miRNAs) class of ncRNAs appears to be absent in some Apicomplexan species including *Plasmodium*. However, an increasing number of studies have described a role of host miRNAs in host-parasite interactions (reviewed by Judice et al., 2016), as host miRNA expression can change after parasite infection and, within this context, suggesting a crucial role for the host immune system signaling.

The incorporation of RNA sequencing data will be extremely important to further work on annotated genes and continue the curation and further characterization of putative genes sets that are being identified in new *P. vivax* isolates under WGS projects. On the other hand, transcript data will indicate the significance of SNPs and increase of copy number variation (CNV) during the hypothesized recent population expansion in *P. vivax* (Mu et al., 2005; Taylor et al., 2013; Cornejo et al., 2015; Parobek et al., 2016). Furthermore, genome-wide transcriptome analysis already contributes to a better understanding of the dynamics of *P. vivax* gene expression by annotating new coding and non-coding transcripts, determining absolute levels of some transcripts, and characterizing the biological importance of UTRs, TSSs and alternative splicing sites (Zhu et al., 2016). Most importantly, it gives an understanding of the role of the transcriptional regulatory machinery and fine-tuned mechanisms that promote the large and diverse population upkeep of *P. vivax*, ability to stand up to selective sweeps, including those pressures caused by malaria control measures (Parobek et al., 2016). Even though only few studies into *P. vivax* transcriptional regulation have been published (Bozdech et al., 2008; Hoo et al., 2016; Zhu et al., 2016), some results suggest that specific biological processes that have enabled the parasite to avoid the traditional malaria control measures might be under tight transcriptional control. For example, early gametogenesis (Bousema and Drakeley, 2011) along with the asexual cycle seems to be achieved by transcriptional repression of an AP2 transcription factor (Yuda et al., 2015; Hoo et al., 2016). Another example is the biological process underlying hypnozoite latent stage formation and activation, suggested to be under epigenetic regulation. Studies using simian hepatocyte cultures report an acceleration of hypnozoite activation when histone methylation is inhibited. Thus, methylation of histones promotes suppression of transcription, and ultimately, maintain hypnozoite dormancy (Barnwell and Galinski, 2014; Dembele et al., 2014). Other example of *P. vivax* transcriptional regulation are related to mechanisms of drug resistance emergence, has shown for differential expression of CRT protein, rather than *pvcrt* gene coding sequence alteration, mediating CQ resistance (Fernandez-Becerra et al., 2009; Melo et al., 2014; Pava et al., 2015).

## *Plasmodium vivax* proteomics and metabolomics

### Parasite and patient serum proteome analysis

In spite of all advances mentioned above, the functions of half of the predicted proteins in *P. vivax* remain unknown. Unsurprisingly, *P. vivax* and *P. falciparum* share a similar metabolic potential with key housekeeping pathways and functions and a range of putative membrane transporters, including the important apicoplast metabolism, essential for the parasite (Carlton et al., 2008a). The apicoplast proteome plays an important role in several major parasite metabolic processes, including some described for *P. falciparum* (Ralph et al., 2004) including isopentenyl diphosphate and iron sulfur cluster assembly, type II fatty acid synthesis and haem synthesis pathways, the latter subdivided between the apicoplast and mitochondria. The localization of several other central pathways is being clarified. For example, it was confirmed that the glyoxalase pathway occurs in the apicoplast, although in *P. vivax*, thiamine pyrophosphate biosynthesis takes place in the cytosol (Baird, 2010).

Although *P. falciparum* studies gave us some indications on the principal features of the *P. vivax* proteome, parasite-specific biology probably involves expression of different gene families. Thus, alternative invasion and host escape pathways still remain largely unknown. Stage-specific analysis of *P. vivax* IDC from natural isolates is extremely challenging due to low parasitemias, characteristically asynchronous parasite populations, and frequent polyclonal infections. In order to overcome the low parasitemia data challenge, some studies have pooled samples from different patients. However, such procedures can potentially increase the assortment of proteins detected, render their identification and association with disease pathogenesis more difficult, and affect the reproducibility of vivax proteomic data and follow up validation studies. Proteome studies are expected to contribute greatly by disclosing proteins expressed in clinical isolates and reveal new unique parasite pathways involved in malaria pathophysiology, leading to identification of new targets for drug and vaccine development.

Efforts are being made to identify potential vivax malaria biomarkers to understand host responses. The earliest proteome study, published in 2009 (Acharya et al., 2009), identified 16 proteins from a single patient infected with *P. vivax* blood-stage parasites (Figure 2). In 2011, the same authors were the first to examine the *P. vivax* proteome directly from a pool of clinical isolates, identifying a total of 153 *P. vivax* proteins, most of them with unknown homology to expressed genes (Acharya et al., 2011). That same year Roobsoong et al. (2011) published the *P. vivax* schizont proteome from multi-patient schizont cultures and enriched samples, where they reported 316 proteins of which 36 were unique to this parasite, and half of these were unique hypothetical proteins. Such proteins could be *P. vivax*-specific targets for diagnosis and treatment, as the four novel *P. vivax* antigens described (Roobsoong et al., 2011). More recently, 238 trophozoite proteins were identified in *P. vivax* strain VCG-1 together with 485 from the *Aotus* host (Moreno-Perez et al., 2014). The in-depth analysis of two *P. vivax* Sal-1 proteomes (*S. boliviensis* monkey iRBCs at the trophozoite stage) allowed the identification of 1375 parasite and 3209 host proteins and their post-translational modifications, mostly N-terminal acetylation (Anderson et al., 2015).

**Figure 5:**
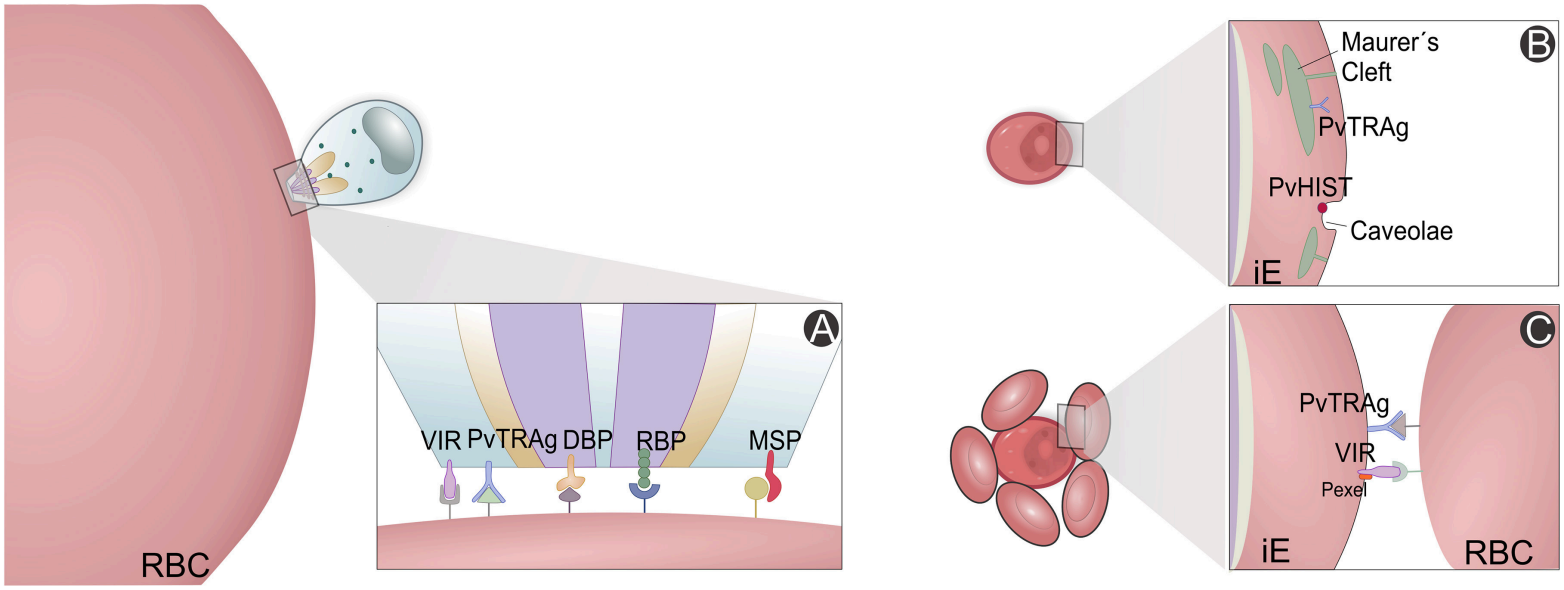
*P. vivax* variant proteins. Slices of the *P. vivax* infected erythrocyte (Pv-iE) are shown with known variant proteins (VIR, PvTRAg, DBP, RBP, PvHIST, MSP) involved in *P. vivax* merozoite invasion **(A)**, transport structures mediating the export of parasite ligands on the surface that promote changes in the host erythrocyte **(B)** and proteins associated with host evasion mechanisms, e.g., the rosetting phenotype **(C)**. RBC, red blood cell; iE, infected erythrocyte; VIR, variable interspersed repeat multigene family proteins; PvTRAg, *P. vivax* tryptophan-rich antigen family proteins; PvHIST, *P. vivax* Plasmodia Helical Interspersed SubTelomeric multigene family proteins; DBP, Duffy binding protein; RBP, reticulocyte binding proteins; MSP, Merozoite surface proteins; Pexel, *Plasmodium* export element protein motif.

High-throughput screening has been used to study *P. vivax* immune proteomes, leading to identification of 44 antigens from a total of 152 protein putative candidates, and characterization and confirmation of rhoptry-associated membrane antigens (PvRAMA) as relevant serological markers of recent exposure to infections (Lu et al., 2014). Furthermore, differences between *P. vivax* and *P. falciparum* antigenic genes were revealed. Most of these highly immunoreactive proteins are hypothetical, but there have been suggestions of their importance for *P. vivax* invasion and evasion mechanisms (Figure 5). For instance, reticulocyte binding protein 2 and RAMA maybe involved in selectivity for invasion of RTs and/or exported protein 1 and 2, histidine-rich knob protein homolog and aspartic protease PM5 for host immune evasion (Lu et al., 2014).

Humoral immunity of vivax malaria patients has also been interrogated. Chen and co-authors cloned and expressed 89 proteins that were screened against sera from vivax malaria patients using protein arrays to show 18 highly immunogenic of which 7 had been already characterized as vaccine candidates and the remaining were uncharacterized (Chen et al., 2010). Later, Ray and colleagues (Ray et al., 2012a,b) identified proteins such as apolipoprotein A1 and E, serum amyloid A and P, haptoglobin, ceruloplasmin, and hemopexin as differentially expressed in uncomplicated malaria patients by serum proteome analysis. Collectively, these results indicate that several physiologically important host pathways have been modulated during parasitic infection including vitamin D metabolism, hemostasis and the coagulation cascade, acute phase response and interleukin signaling, and the complement pathway (Ray et al., 2012a).

### Host-parasite metabolic pathways

To uncover pathway modulation occurring during uncomplicated (Ray et al., 2012a,b) vs. severe vivax malaria (Ray et al., 2016), researchers are going further into clinicopathological analysis and proteomic profiling. Recently, human serum proteome comparative studies were published (Ray et al., 2016) showing different levels of parasitemia for severe and non-severe infections, as well as during the acute and convalescent phases of the infection (Ray et al., 2017). The involvement of the acute phase response was confirmed, including acute phase reactants or proteins, cytokine signaling, oxidative stress and anti-oxidative pathways, muscle contraction and cytoskeletal regulation, lipid metabolism and transport, complement cascades, and several coagulation and hemostasis associated proteins in malaria pathophysiology (Ray et al., 2016, 2017). However, alterations in the blood coagulation cascade, as reported for severe falciparum malaria, were not identified for severe vivax malaria (Ray et al., 2016). Moreover, these proteomic studies allowed the identification of prospective new host markers, such as the differentially expressed proteins superoxide dismutase, ceruloplasmin, vitronectin, titin, and nebulin. Furthermore, they confirmed the already investigated apolipoprotein A1 and E, serum amyloid A and haptoglobin markers. Those are important for future improved diagnosis, discrimination of other infections, as well as disease progression and shift toward severe clinical manifestations, response to new therapies and outcome prediction (Ray et al., 2016). Analysis of a recent longitudinal cohort of vivax malaria patients revealed that some serum levels of proteins such as haptoglobin, apolipoprotein E, apolipoprotein A1, carbonic anhydrase 1, and hemoglobin subunit alpha, revert to baseline levels under treatment, but others did not show the same behavior in the convalescent phase of the infection (Ray et al., 2017), which might reflect the effect on the host of different parasitemias present. Serum proteome results also prompt the malaria community for a clearer definition of severity parameters for *P. vivax* malaria and show a marked difference between the pathogenesis of infection caused by different *Plasmodium* parasites (Ray et al., 2016).

The panorama emerging from all malaria proteome studies is that a great fraction of proteins identified until now are uncharacterized and of unknown functions, alongside with several housekeeping proteins. However, other proteins of major metabolic pathways for parasite survival show us that we are in a good position to better understand immune evasion and host cell invasion mechanisms within the pathophysiology of vivax malaria. These pathways comprise metabolism (glycolysis, hemoglobin digestion, nucleic acid synthesis) and cellular invasion (binding proteins, protein synthesis, modification, and degradation) (Figure 5A). Intracellular transport, translocation and presentation of variable antigen proteins (Figure 5B), which promote erythrocyte modification (Figure 5C), have been carefully analyzed, in particular proteins directly related to drug resistance. All chaperonin complexes, some involved in heat shock, oxidative stress and other counteractive responses, have been identified (Acharya et al., 2009, 2011; Chen et al., 2010; Roobsoong et al., 2011; Ray et al., 2012a,b, 2016; Lu et al., 2014; Moreno-Perez et al., 2014; Anderson et al., 2015).

Although there was evidence of transcription for genes encoding almost all *P. vivax* proteins identified, no correlation was seen between levels of mRNA transcripts and the corresponding protein expression (Bozdech et al., 2008; Westenberger et al., 2010; Bautista et al., 2014; Moreno-Perez et al., 2014; Anderson et al., 2015; Parobek et al., 2016). Integrating genomic and transcriptomic information is paving the way for systems biology approaches. For instance, chokepoint analysis has the potential to uncover enzymes predicted to have only one substrate or product, thus pushing the way to new drug target discoveries. Lists of compounds that could target each chokepoint are being generated using bioinformatics tools from “omics” data analysis.

Analysis of the hypnozoites proteome faces great experimental hurdles. A list of candidate genes includes all homologs of other dormancy genes previously discovered in other organisms (Carlton et al., 2008a). An *in vitro* system for culturing liver stages of this parasite (Mazier et al., 1984; Hollingdale et al., 1985; Sattabongkot et al., 2006; Cui et al., 2009) and *in vivo* experimental models are currently under development and will be of importance to approach hypnozoite proteomics. Taken together, blood proteome studies shed light on vivax malaria pathogenesis and pave the way for future integrated multi-omics investigations. However, such an approach has to be interpreted in light of host-parasite interaction, where some reported alterations might be host or parasite related and/or the result of cumulative effects between both parties. *Ex vivo* specific functional assays may provide further insights.

## *Plasmodium vivax* genetic and transcriptomic diversity, host-parasite dynamics, and disease control

The first *P. vivax* genetic diversity reports were based on a few loci. Researchers relied on the amplification of a small set of polymorphic antigens to assess the genetic diversity of *P. vivax*. In general, the circumsporozoite protein (CSP), the merozoite surface proteins (MSP), the apical membrane antigen (AMA-1) (Mueller et al., 2002; Cui et al., 2003; Kim et al., 2006; Aresha et al., 2008; Moon et al., 2009; Zakeri et al., 2010), Duffy binding protein (DBP) (Cole-Tobian and King, 2003) and mitochondrial DNA were analyzed (Jongwutiwes et al., 2005; Cornejo and Escalante, 2006) and brought forward the polyclonal nature of *P. vivax* natural infections. Feng et al. published the first comparative population genomics study between *P. falciparum* and *P. vivax*, revealing shared macroscale conserved genetic patterns, but with *P. vivax* populations showing a highly polymorphic genome for which the degree of diversity depends on the gene context. Thus, coding regions are more conserved than intergenic ones, and central chromosomal regions more than (sub)telomeric, indicative of a purifying selection. This implies an ancient evolutionary history shaped by distinct selection pressures (Feng et al., 2003; Chang et al., 2012). Genes encoding transcription factors (type AP2) and ABC transporters associated with multidrug resistance showed higher dN/dS rate (Dharia et al., 2010; Parobek et al., 2016). The genetic variability seen for *P. vivax* transcription factors (Hoo et al., 2016) could indicate that malaria parasites evolve by changing and/or expanding their transcription factor DNA binding domains in response to drug administration and host immune challenges (Dharia et al., 2010; Parobek et al., 2016).

Several studies have highlighted the great degree of diversity, which is shared between *P. vivax* samples of different geographic locations at the population level, strongly suggesting that recent evolutionary selective pressures are currently acting upon some genes at a few loci, mainly those related with drug resistance (Mu et al., 2005; Imwong et al., 2007a; Gunawardena et al., 2010; Neafsey et al., 2012; Lin et al., 2013). Compared to *P. falciparum* natural isolates from similar geographical regions (Cheeseman et al., 2016), results exposed a greater genetic diversity in *P. vivax* natural isolates, both for SNP and microsatellites (Karunaweera et al., 2008; Neafsey et al., 2012; Orjuela-Sanchez et al., 2013; Taylor et al., 2013; Barry et al., 2015; Winter et al., 2015; Brazeau et al., 2016; Friedrich et al., 2016; Hupalo et al., 2016; Pearson et al., 2016). While microsatellite marker analysis has revealed the genetic diversity of *P. vivax* infections, they may provide over- or underestimates. This can be attributed to their unstable nature in comparison to SNPs, especially because of the smaller number and/or less informative use of these markers. Therefore, *P. vivax* high density tiling microarrays (Dharia et al., 2010), WGC sequencing (Bright et al., 2012) and WGS (Bright et al., 2012; Chan et al., 2012; Neafsey et al., 2012; Hester et al., 2013; Winter et al., 2015; Hupalo et al., 2016; Pearson et al., 2016) are being used to find informative SNPs. Whole genome analysis of genetic diversity between five *P. vivax* natural isolates (Neafsey et al., 2012) and the Sal-1 strain (Carlton et al., 2008b) has revealed shared gene diversity, but also significant numbers of unique SNPs for each isolate (Hester et al., 2013; Cornejo et al., 2015; Hupalo et al., 2016). Genomic and transcriptomic sequencing of several patient *P. vivax* isolates resulted in drug resistance phenotype identification (Bright et al., 2012; Mideo et al., 2013; Lin et al., 2015), emergence detection, transmission rates follow-up, as well as the identification (also through proteomics) of rapidly evolving antigens (Boyd and Kitchen, 1937; Cornejo et al., 2015; Winter et al., 2015; Hupalo et al., 2016; Parobek et al., 2016; Pearson et al., 2016). Genomics data suggest that evolutionary pressures acted upon and currently shape several *P. vivax* loci associated with invasion of RBC (Figures 5A,B), host and vector immune evasion mechanisms (Figures 5B,C) and antifolate drug resistance, among others (Imwong et al., 2003; Korsinczky et al., 2004; Dharia et al., 2010; Flannery et al., 2015; Winter et al., 2015; Hupalo et al., 2016; Pearson et al., 2016), which could support target discovery (Carlton et al., 2013; Cornejo et al., 2015; Hupalo et al., 2016; Pearson et al., 2016). For instance, WGS of two patient isolates from Madagascar allowed the identification of recently arisen SNPs present in the DBP region II (Chan et al., 2012). The detection of *pvdbp* gene duplication (Menard et al., 2013; Hupalo et al., 2016; Pearson et al., 2016) suggests that this gene is under strong positive selection through influence of the human host, and is rapidly evolving in response to the Duffy- allele present in most of sub-Sharan African population, where *P. vivax* is believed to have originated (Miller et al., 1976; Culleton et al., 2011; Liu et al., 2014). Surprisingly, similar duplications at high frequency have been recently seen in other geographic locations where almost all individuals are Duffy+ (Howes et al., 2011; Pearson et al., 2016). More recently, Auburn and colleagues reported amplification breakpoints for the multidrug resistance 1 gene (*pvmdr1*) (Auburn et al., 2016b). Thus, *P. vivax* is considered within an evolutionary neutral model, possibly the outcome of repeated gene duplications (population size expansion) of the ancestral lineage during the course of evolution, or that several sub-populations are present (Parobek et al., 2016). Multi-species WTS data suggests that variability in non-coding sequences of genes but other features such as transcription factors, upstream bidirectional promoter regions, post-transcriptional control including epigenetic regulation, chromatin remodeling events or ncRNAs may have a greater impact on transcription modulation across the *Plasmodium* spp. (Hoo et al., 2016).

With few exceptions verified in small-scale studies (Winter et al., 2015), *P. vivax* natural infections are almost always polyclonal (Parobek et al., 2016). However, some haplotype reconstruction studies have reported that 2–4 strains account for the majority of *P. vivax* gDNA, set against a situation of several different strains equally abundant in one patient (Chan et al., 2012; Neafsey et al., 2012; Winter et al., 2015; Friedrich et al., 2016; Pearson et al., 2016). For instance, deep genome sequencing of more than 200 clinical Asian-Pacific samples confirmed the complex genetic structure of *P. vivax* populations showing variations both in the number of dominant clones in individual infection, but also in their degree of relatedness and inbreeding (Pearson et al., 2016). Still, this remarkable polyclonality can have several origins: parasite infection could originate from a single meiosis event, from multiple infections with unrelated parasites as a consequence of several mosquito bites, or from a combination of a relapse and a new infection event, all of them having implications on parasite population diversity (Lin et al., 2015). Microsatellite marker identification and WGS have allowed the characterization of relapse infections as the result of heterologous hypnozoite activation (Imwong et al., 2007b; Carlton et al., 2008a; Restrepo et al., 2011; de Araujo et al., 2012; Bright et al., 2014; Lin et al., 2015). Furthermore, hypnozoites present hidden in the hosts function as a reservoir and a dynamic and continuous source and flow of genetic diversity in the present *P. vivax* population (White, 2011; Neafsey et al., 2012; Menard et al., 2013; White et al., 2016).

Even though WGS allows determination of informative SNPs, it lacks sensitivity to detect low frequency CNVs present within a polyclonal infection context. The tailored and more affordable *P. vivax* SNPs barcode project (Baniecki et al., 2015), transcriptome profiling microarrays (Boopathi et al., 2016) and other breakpoint-specific PCR approaches (Auburn and Barry, 2017) can be used as important tools to not only discriminate *P. vivax* infections and their origin, but also to deliver information of its population dynamics of transmission (Friedrich et al., 2016). Additionally, they are helping to characterize and discern between recrudescence, relapses (Lin et al., 2015), *de novo* infections (Friedrich et al., 2016) and monitoring drug resistance emergence (Auburn et al., 2016b; Brazeau et al., 2016). As for *P. falciparum* (Cheeseman et al., 2016), this greater genetic/expression diversity may provide *P. vivax* with a wide range of alternative paths for successful host erythrocyte invasion (Figures 5A,B) and immune system evasion (Figure 5C), thus enhancing its capacity of adaptation and survival (Cornejo et al., 2015). In addition, the production of an effective vivax malaria vaccine is hampered by the existence of such huge diversity of antigens concentrated in invasion and immune evasion related genes (Neafsey et al., 2012; Winter et al., 2015; Hupalo et al., 2016; Pearson et al., 2016) in combination with the absence of allelic exclusion and clonal interference mechanisms (Fernandez-Becerra et al., 2005).

## *Plasmodium vivax* omics outlook: achievements, gaps, and future prospects

During the last 10 years, several P. vivax sequencing projects within the three big omics branches, genomics, transcriptomics and proteomics, have greatly contributed with insights into the parasite's biology (Table 2). The lack of a *P. vivax in vitro* long-term culturing system is the root of all limitations for its study. Nevertheless, we are living in times of rapid development, expansion and application of high-throughput and highly sensitive technology tools that were absurdly expensive only a few years ago. Exciting progress is being made in single-cell (Nair et al., 2014) and single-molecule sequencing, bringing prospects of complex problem-solving capacity that were previously unthinkable. The development of methodologies such as CRISPR/Cas9 system for genome editing opens new possibilities to understand and further explore gene expression and regulation of malaria parasites. The application of such tools to *in vitro* cell or animal experimental models may help us explore further some aspects of *P. vivax* biology and the pathophysiology of disease (Table 3). Specifically, the development, establishment and validation of *in vitro* cell culture and animal experimental models (as the published Mazier et al., 1984; Hollingdale et al., 1985; Sattabongkot et al., 2006; Cui et al., 2009; Mikolajczak et al., 2015; Mehlotra et al., 2017 reports) to support *P. vivax* growth would overcome the current number of parasites available for projects in all omics branches. Clearly, this would positively impact on our understanding of several molecular mechanisms taking place in the parasite during cell invasion, development, stage transition, host immune system evasion and environmental stress responses (Table 3).

**Table 2:** *Plasmodium vivax* omics achievements and ongoing research efforts.

**Achievements**	**Ongoing research**
***P. VIVAX*** **GENOMICS**	
Monkey-adapted *P. vivax* strains WGS *P. vivax* Sal-1 reference genome and recent publication of the new *de novo* assembled *P. vivax* P01 reference genome Direct WGS of *P. vivax* isolates distributed worldwide for genetic diversity characterization and sequence variation identification to understand the current selective pressures, especially those resulting from malarial control measures Comparative genome-wide studies between different *Plasmodium* spp.	Monkey *P. vivax* strain adaptation systems to study diversity, population dynamics, parasite multi-stage biology and host interaction Enhanced curation of *P. vivax* P01 reference genome: coding and non-coding sequences, regulatory motifs and variants characterization WGS and CGA within *P. vivax* isolates from different high and/or low transmission endemic areas, and from resistance emergence hot spot areas WGS of *Plasmodium* spp. closely related to *P. vivax* for parasite biology studies
***P. VIVAX*** **TRANSCRIPTOMICS**	
Transcriptome portrayal of *P. vivax* IDC and sporozoites by microarray, and recent more complete and sensitive reports by RNA-seq *P. vivax* IDC and WTS of patient isolates The structural analysis of *P. vivax* transcripts, where multiple TSSs, long 5'UTRs and alternative splicing events are observed suggest multiple protein isoforms expressed by the parasite; Transcript structure abundance and characterization, together with identification of ncRNAs hint at a strong and tight mechanism of transcription regulation control	Expression profile analysis aids identification of groups of genes which may have important roles in parasite development, invasion, host immune system evasion and drug resistance emergence mechanisms, and their diversity across several *Plasmodium* spp. Impact of transcript structural features on expression observed: NATs, multiple TSSs clusters, long 5′UTRs and alternative splicing events leading to multiple protein isoforms produced Studies on mechanisms of transcription regulation: chromatin remodeling, transcriptional run-through, bidirectional and antisense specific promoters
***P. VIVAX*** **PROTEOMICS AND METABOLOMICS**	
Proteomic MS datasets representing *P. vivax* trophozoites and schizonts to identify stage-specific expression of parasite proteins and protein isoform Several serum proteome studies from clinical blood samples from infected patients Identification of highly immunogenic proteins as new molecular targets aiming for drug and vaccine design; Some important parasite pathways with roles in host-parasite interaction have been identified, e.g., those related with parasite metabolism, host cell invasion, intracellular transport, translocation and presentation of variable antigen proteins and stress response and drug resistance	Protein structure and function characterization, identification of post-translational modifications and new isoforms Comparative proteome analysis between severe and uncomplicated vivax malaria cases, uncomplicated vivax malaria patients before and after treatment, and high vs. low parasitemia isolates Drug and vaccine design and test against molecular targets already identified as highly immunogenic proteins Description and study of protein participation in parasite metabolic pathways involved in host-parasite interactions

*WGS, whole genome sequencing; WTS, whole transcriptome sequencing, RNA-seq, RNA sequencing; CGA, comparative genome analysis; IDC, intraerythrocytic developmental cycle; UTR, untranslated region; ncRNAs, non-coding RNAs; NATs, natural antisense transcripts; MS, mass spectrometry*.

**Table 3:** *Plasmodium vivax* omics challenges and future progress in a systems biology setting.

**Research challenges**	**Future perspectives**
**BIOLOGY: EXPERIMENTAL ASSAYS AND MODELS FOR NEW BIOLOGICAL INSIGHTS**	
*P. vivax* IDC *in vitro* long-term culturing system Primate, murine and other *P. vivax* animal models to support stage-specific and stage-transition studies Studies with closely related *Plasmodium* spp. other than *P. falciparum*, in general primate and murine, for which *in vitro* or *in vivo* culture are available: investigation into similarities and differences on the nature of molecular mechanisms taking place in the parasites	Search for new alternative *in vitro* cell line cultures to support culture of *P. vivax* IDC Use of new and reliable *P. vivax* animal models, of easy handling and availability, for host-parasite biology studies, Explore *Plasmodium* spp. closer to *P. vivax* as a way to understand better its biology, principally about liver (hypnozoites) and sexual (gametocytes) stages, and also to test for drug efficacy and drug resistance emergence and mechanisms
**NGS: METHODOLOGY DEVELOPMENT AND VALIDATION OF NEW DATA REPORTS**	
Sequencing technology that can deal better with the current field isolate characteristics Monitor with precision the current *P. vivax* genetic diversity through a WGS easy-to-use platform WTS projects to characterize transcriptomic diversity on different parasite stages in different field isolates Progress on MS proteome screens on highly stage-synchronized parasite samples Experimental approaches that capture the interactions between DNA, RNA and protein through their combinations as to grasp the regulatory mechanisms suggested to underlie the distinct *P. vivax* biology Investigate the *Plasmodium*-like species genome, transcriptome and proteome sequence composition, expression profiles and functional properties	Develop improved sequencing technology of higher sensitivity and accuracy for low-input and heavily contaminated biological samples to sequence the parasite genome, transcriptome or proteome WGS projects on *P. vivax* isolates as to real-time screen the genetic variation within and between different endemic regions linked to drug resistance emergence WTS projects to extend the study of transcript expression patterns, levels and structure throughout different *P. vivax* stages Proteome studies with the aim to identify highly immunogenic proteins, their functions, detect new protein isoforms and characterize post-translational modifications Epigenomic exploration Extensive orthology projects on close related *Plasmodium*-like species and parasite samples derived from *in vitro* and *in vivo* experimental studies
**COMPUTATION: PREDICTIVE SOFTWARE DATA ANALYSIS FOR NEW HYPOTHESIS-DRIVEN QUESTIONS**	
Develop better way to assembly and annotate highly repetitive and variable regions (e.g., (sub)telomeric and centromeric) for coding and non-coding sequences and variants, regulatory motifs and protein isoforms Increase comparative genome-wide studies between different *Plasmodium* spp. and other experimental datasets as the way to associate and infer *P. vivax* biological processes Real-time characterization of *P. vivax* populations and drug resistance hot spots identification Transcript sequence and structural information: chromatin remodeling (epigenomics), transcriptional run-through, bidirectional and antisense specific promoters Identify and link protein functions into specific parasite pathways to further characterize host-parasite interactions	Improve the current assembly and annotation of *P. vivax* P01 reference genome and transcriptome, using third NGS platforms (e.g., nanopore and PacBio) to aid the assembly of highly repetitive and variable regions Integrate data to better understand processes governing parasite development, host invasion and immune system evasion, and drug resistance mechanisms Use computational modeling for analysis of the genetic diversity of the dynamic endemic *P. vivax* populations and software for data analysis to explore current selective pressures and predict drug resistance emergence and the outcomes of malarial control measures Transcriptomic and epigenomic studies to understand transcriptional regulation control Uncover network building-blocks involved in metabolism, host cellular invasion, intracellular transport, translocation and presentation of variable antigen proteins and stress response

*IDC, intraerythrocytic developmental cycle*.

The extensive and detailed omics datasets that are continuously made available by several research groups, under which technical and experimental progress has and is being achieved brings forward an old problem: how to analyze and integrate large and complex information within and between datasets in a standard and easy-to-share platform, accessible by all. Several initiatives, such as the comparative databases PlasmoDB (Bahl et al., 2003), support malaria research communities by offering computational biology training courses. Such platforms should be continuously developed with the most recent powerful bioinformatics tools and information combined with suitable mathematical modeling and epidemiologic analysis. Mainstream bioinformatics institutes around the world are providing expert support to the *Plasmodium* research community, critically needed given the extraordinary challenges posed by the complex nature of the *P. vivax* genome and population genetics. Worldwide, major efforts must be taken by the entire community in order to establish some criteria for dealing with data collection, analysis and annotation, so that it can be easily accessed and fully explored in order to design adequate interventions to ever changing conditions, focusing on the control, elimination and prevention of malaria.

## Author contributions

CB and LA contributed to literature review and writing of the manuscript. CB and AK were responsible for the original figure concept and design. LA, PS, and FC helped with critical revision of the manuscript for intellectual content. All authors read and approved the final manuscript.

### Conflict of interest statement

The authors declare that the research was conducted in the absence of any commercial or financial relationships that could be construed as a potential conflict of interest.

## Abbreviations

ACT: Artemisinin based Combination Therapy
AMA: Apical Membrane Antigen
apDNA: apicoplast DNA
bp: base pairs
CGA: Comparative Genome Analysis
ChIP-seq: chromatin immunoprecipitation (ChIP) DNA sequencing
CQ: Chloroquine
CNV: Copy Number Variation
CSP: Circumsporozoite Protein
CVC: caveola-vesicle like complexes
DBP: Duffy Binding Protein
dN: non-synonymous substitution rate
dS: synonymous substitution rate
ETRAMP: Early Transcribed Membrane Protein
gDNA: genomic DNA
IDC: Intraerythrocytic Development Cycle
iRBC: infected Red Blood Cell
Kb: kilobases
Mb: megabases
miRNA: micro RNA
mRNA: messenger RNA
MS: Mass Spectrometry
MSP: Merozoite Surface Protein
MudPIT: multidimensional protein identification technology
mtDNA: mitochondrial DNA
NAT: Natural Antisense Transcript
ncDNA: nuclear DNA
ncRNA: non-coding RNA
NGS: Next Generation Sequensing
nt: nucleotide
Pexel: *Plasmodium* export element protein motif
PQ: Primaquine
PST-A: lysophospholipase
PvHIST, *P. vivax*: Plasmodia Helical Interspersed SubTelomeric multigene family proteins (also named as Pf-fam-b) and RAD protein (Pv-fam-e)
PvTRAg, *P. vivax*: tryptophan-rich antigen family proteins, also named as Pv-fam-a and tryptophan-rich antigens
PvSTP-1: *P. vivax* STP1 protein
RBC: Red Blood Cell
RBP: Reticulocyte Binding Protein
RAMA: Rhoptry-Associated Membrane Antigen
RNA-seq: RNA sequencing
RT: Reticulocytes
SNP: Single Nucleotide Polymorphism
TAP: Transcriptional Associated Protein
TLR: Toll-like Receptor
TSS: Transcription Starting Site
UTR: Untranslated Regions
VIR: Variable Interspersed Repeat
WGC: Whole Genome Capture
WGS: Whole Genome Sequencing
WGSS: Whole Genome Shotgun Sequencing
WHO: World Health Organization
WTS: Whole Transcriptome Sequencing
1D LC-MS/MS: one-dimension liquid chromatography–mass spectrometry.
